# Management of Gastroenteritis in an AIDS Patient With a History of Multiple Secondary Infections and Psychiatric Disorders

**DOI:** 10.7759/cureus.69188

**Published:** 2024-09-11

**Authors:** Dawn-Noella C Ikechi-Konkwo, Keren-Happuch A Ikechi-Konkwo, Manon Djomani

**Affiliations:** 1 Department of Psychiatry, Jackson Memorial Hospital, Miami, USA; 2 Department of Medicine, American University of the Caribbean, Cupecoy, SXM

**Keywords:** bipolar, depression, integrative medicine, medical compliance, poor adherence, schizophrenia

## Abstract

This case report explores the challenges associated with the management of chronic medical diseases in patients with psychiatric disorders. The patient is a 36-year-old female patient diagnosed with AIDS, multiple secondary infections, major depressive disorder, anxiety, and polysubstance abuse. The simultaneous occurrence of both physical and mental health conditions presents unique obstacles in managing chronic medical diseases. The goal of this report is to discuss the patient's medical history, psychosocial factors, interventions, and outcomes and provide insights for future patient care. This report will also illuminate the relationship between psychiatric disorders and diminished health maintenance.

## Introduction

Human immunodeficiency virus (HIV) is predominantly a sexually transmitted infection that destroys CD4+ T lymphocyte cells, while acquired immunodeficiency syndrome (AIDS) is the final disease stage of the original viral infection. A diagnosis of AIDS is based on a CD4+ T lymphocyte count of <200 and/or the presence of an AIDS-defining illness. The rapid progression of HIV to AIDS can be due to poor treatment adherence [1]. Research has shown that some psychiatric disorders such as depression, schizophrenia, and bipolar disorder can lead to a decline in compliance with medications and patient follow-up [2]. The conditions feature hallmark characteristics that may play a role in the patient’s poor adherence; they can constitute diminished reasoning and poor insight into the patient’s diagnoses. Major depressive disorder (MDD), in particular, is one of the most common mental health disorders present in patients with HIV and is often linked with decreased treatment adherence and compliance [2-5]. Individuals with psychiatric disorders often face barriers to accessing and adhering to medical care, leading to the exacerbation of chronic diseases. This report delves into the reasons behind the patient's diminished treatment compliance, emphasizing the influence of MDD, anxiety, and polysubstance abuse. It will also work to advocate for holistic care and integrated healthcare approaches to optimize patient outcomes.

## Case presentation

The patient is a 36-year-old woman with a past medical history of Monkeypox, COVID-19, HIV, and syphilis. She also has a past psychiatric history of MDD, anxiety, and polysubstance abuse. She was diagnosed with depression and anxiety a year prior and denied taking any medication for either. She presented to the behavioral hospital on an involuntary admission for two weeks. The patient was brought in by police after she was found wandering and incoherent on the street. A drug test on admission was positive for marijuana and cocaine. The patient admitted to regular alcohol use. A week after admission, the patient has been complaining of foul-smelling, watery diarrhea and severe, diffuse abdominal pain for the past one week. She was seen by the hospitalist on duty in the behavioral hospital. She denied any nausea, vomiting, or other related symptoms. The patient was then diagnosed with Gastroenteritis. The patient is homeless and admitted to being aware of her HIV diagnosis for two years. She also stated that she had not been to a physician for “a while”. The patient states that she is unaware of any family history. She was prescribed Biktarvy when she was first diagnosed but couldn’t recall the specific dosage or the last time she took her medication. The patient also stated that she’d had a self-resolving bout of similar symptoms a few months ago.

On admission, her vital signs were as follows: blood pressure of 97/79 mmHg, heart rate of 87 beats per minute, temperature of 97.7 Fahrenheit, respiratory rate of 18 breaths per minute, and oxygen saturation of 100%. The patient was awake, alert, and oriented but in obvious distress. No psychometric tests were conducted. Physical examination revealed no abnormalities: Abdomen was soft and non-tender to palpation. She had a negative pregnancy test and had not received any recent imaging. Laboratory values revealed that her CD4 count was 2, bicarbonate was 21, and chloride was 111 (Table 1). These levels may be due to a loss of bicarbonate in the stool. A blood culture revealed a positive TOXO antibody which signifies a current or previous toxoplasmosis infection and a COVID-19 test was positive. All other lab results were within normal limits.

**Table 1:** Laboratory values: bicarbonate, chloride, and CD4+ T-lymphocyte count

Variables	Patient values	Reference range
Bicarbonate	21 mEq/L	22–28 mEq/L
Chloride	111 mEq/L	95–105 mEq/L
CD4+ T-lymphocyte count	2/mm^3^	≥500/mm^3^

The patient was diagnosed with AIDS due to her CD4 count<200 and AIDS-defining illness. The complex treatment plan for this patient included management of both psychiatric and medical diagnoses. Upon admission, the patient was given diphenhydramine, lorazepam, and haloperidol for agitation. While on admission, her acute gastroenteritis was treated with ciprofloxacin and oral hydration, with imodium for symptomatic relief. Her AIDS treatment required adherence to her current regimen of Biktarvy daily [6,7], with the addition of Bactrim prophylaxis daily. For management of her psychiatric diagnoses of MDD and anxiety, the patient was started on Lexapro and hydroxyzine pamoate; respectively. Due to the patient's alcohol abuse, which may have caused malnutrition, she was given thiamine, folic acid, and a daily multivitamin. She also received a nicotine patch as needed to aid with smoking cessation and trazodone as a sleep aid (Table 2).

**Table 2:** Medications during admission BID: twice a day; IM: intramuscular; Q6H: every six hours; Q2H: every two hours; PRN: as needed; mg: milligrams; mL: milliliters.

Medication	Dosage
Bactrim DS	800mg-160mg 1 tablet, ORAL, DAILY
Biktarvy	50 mg- 200 mg- 25 mg 1 tablet, ORAL, DAILY
Ciprofloxacin	500 mg= 1 tablet, ORAL, BID
Diphenhydramine	50 mg= 1 mL, IM, Q6H, PRN
Folic acid	1 mg= 1 tablet, ORAL, DAILY
Haloperidol	5 mg= 1 mL, IM, Q6H, PRN
Hydroxyzine pamoate	50 mg= 1 capsule, ORAL, Q6H, PRN
Imodium A-D	2 mg= 1 capsule, ORAL, Q6H, PRN
Lexapro	20 mg= 1 tablet, ORAL, DAILY
Lorazepam	2 mg= 1 mL, IM, Q6H, PRN
Multivitamin	1 tablet, ORAL, DAILY
Nicotine patch	Q2H, PRN
Thiamine	200 mg= 2 tablets, ORAL, DAILY
Trazodone	75 mg= 1.5 tablets, ORAL, PRN

During hospitalization, she received group therapy along with other patients four times a week. On day 12 of admission, the patient's symptoms of diarrhea and abdominal pain were resolved. The patient was discharged from the behavioral hospital once her two-week hold was completed. She was given a prescription for continuation of Lexapro and hydroxyzine pamoate, at the same dosage, to manage her psychiatric diagnoses and Bactrim for continued prophylaxis against AIDS-defining illnesses. Prior to discharge, she was offered the help of a social worker in housing placement in a local rehab/shelter and a one-week follow-up. Unfortunately, upon discharge, the patient was lost to follow up. The patient’s eventual outcome is unknown.

## Discussion

The major challenges allowing for progression of this patient’s condition to AIDS include her homelessness, prior mental health diagnoses, and healthcare access. Her paralleled engagement in acute healthcare without adherence to long-term treatment regimens may indicate a lack of understanding regarding her diagnosis and dosage, a more potent non-adherence risk when coupled with her other social determinants (homelessness, loss to follow-up, and stigmatized diagnoses). In such cases, a further understanding is needed of the effects of the individual’s MDD and anxiety diagnoses on her memory, behavior, and comprehension [4,5]. In any case, however, physicians must understand that critical components of patient-centered care include evaluation of the patient’s access to prescription medication, comprehension of the treatment plan, in-built hindrances to retention and follow-up, and understanding of their present diagnosis with special knowledge of the noted diagnosis’ relationship to preexisting conditions [6,7]. 

Present literature indicates that proper comprehension of diagnoses is demonstrated by a knowledge of their relationship to other conditions, and the unambiguous effects of the treatment plan [4]. These promote adherence, especially when addressing the origins and negative effects of various social determinants, and can be evaluated with the teach-back method. Assuring proper adherence relies heavily on ensuring that a patient can vocally relay the crux of their diagnosis and treatment plan after face-time with their physician, as well as addressing external socioeconomic hindrances such as treatment access.

## Conclusions

True patient-centered care involves consideration of all detrimental and common effects of a patient’s comorbidities and a consideration of their other health determinants, socioeconomic status, for example. The efficacy of a physician in situations requiring both long and short-term patient care is contingent on the physician’s ability to examine, account for, and appreciate every facet of the individual directly and indirectly linked to their presentation as a patient. In this case and many others, without adequate management of mental illnesses, treatment of chronic medical conditions may have been delayed, resulting in the patient's degression into unwanted outcomes. Through an integrated approach, one which considers socioeconomic hindrances to care and inadequate treatment of psychiatric illnesses, medical diagnoses can be addressed more efficiently.
